# Dietary pattern transitions, and the associations with BMI, waist circumference, weight and hypertension in a 7-year follow-up among the older Chinese population: a longitudinal study

**DOI:** 10.1186/s12889-016-3425-y

**Published:** 2016-08-08

**Authors:** Xiaoyue Xu, Julie Byles, Zumin Shi, Patrick McElduff, John Hall

**Affiliations:** 1 Priority Research Centre for Gender, Health and Ageing, School of Medicine and Public Health, Hunter Medical Research Institute, University of Newcastle, Newcastle, Australia; 2 Centre for Clinical Epidemiology and Biostatistics, School of Medicine and Public Health, Hunter Medical Research Institute, University of Newcastle, Newcastle, Australia; 3 School of Medicine, University of Adelaide, Adelaide, Australia

**Keywords:** Dietary pattern, Body mass index, Waist circumference, Hypertension, Older people

## Abstract

**Background:**

Few studies explored the effects of nutritional changes on body mass index (BMI), weight (Wt), waist circumference (WC) and hypertension, especially for the older Chinese population.

**Methods:**

By using China Health and Nutrition Survey 2004-2011 waves, a total of 6348 observations aged ≥ 60 were involved in the study. The number of participants dropped from 2197 in 2004, to 1763 in 2006, 1303 in 2009, and 1085 in 2011. Dietary information was obtained from participants using 24 hour-recall over three consecutive days. Height, Wt, WC, systolic and diastolic blood pressure were also measured in each survey year.

The dietary pattern was derived by exploratory factor analysis using principal component analysis methods. Linear Mixed Models were used to investigate associations of dietary patterns with BMI, Wt and WC. Generalized Estimating Equation models were used to assess the associations between dietary patterns and hypertension.

**Results:**

Over time, older people’s diets were shifting towards a modern dietary pattern (high intake of dairy, fruit, cakes and fast food). Traditional and modern dietary patterns had distinct associations with BMI, Wt and WC. Participants with a diet in the highest quartile for traditional composition had a β (difference in mean) of −0.23 (95 % CI: −0.44; −0.02) for BMI decrease, β of −0.90 (95 % CI: −1.42; −0.37) for Wt decrease; and β of −1.57 (95 % CI: −2.32; −0.83) for WC decrease. However, participants with a diet in the highest quartile for modern diet had a β of 0.29 (95 % CI: 0.12; 0.47) for BMI increase; β of 1.02 (95 % CI: 0.58; 1.46) for Wt increase; and β of 1.44 (95 % CI: 0.78; 2.10) for Wt increase. No significant associations were found between dietary patterns and hypertension.

**Conclusions:**

We elucidate the associations between dietary pattern and change in BMI, Wt, WC and hypertension in a 7-year follow-up study. The strong association between favourable body composition and traditional diet, compared with an increase in BMI, WC and Wt with modern diet suggests that there is an urgent need to develop age-specific dietary guideline for older Chinese people.

**Electronic supplementary material:**

The online version of this article (doi:10.1186/s12889-016-3425-y) contains supplementary material, which is available to authorized users.

## Background

China has become an ageing society. The proportion of older people is estimated to increase rapidly from 2000 to 2035, with a predicted one in four people aged 60 or above by 2035 [1]. This change in age structure has an impact on the increasing prevalence of non-communicable diseases(NCDs), especially for people in the old age group [2]. In addition, the prevalence of overweight and obese people in all age groups has increased dramatically in the past decade in China [3].

Obesity is not only a chronic condition in itself, but is also an important biological risk factor for NCDs. Diet has been widely identified as a factor in the prevention of obesity [4]. Aging is associated with a decline in a number of physiological functions, which can impact nutritional status, such as reduced lean body mass, a resultant decrease in basal metabolic rate and chronic illness [5]. Although healthy eating to promote healthy ageing is extremely important, research on dietary changes with age, and exploration of the association between diet and NCDs for the older population, are extremely scarce [6].

In China, the number of studies on the association between dietary pattern and NCDs is increasing. However, most of these follow a cross-sectional study design [7–9], with the main focus on children and adolescents [7, 8]. We previously reported the associations between dietary pattern and obesity, as well as hypertension, among older Chinese using a cross-sectional study design. We found a negative association between rice-based traditional dietary pattern and obesity, and a positive association between processed meat/fast food based modern dietary pattern and obesity [3]. Rice-based traditional dietary pattern was negatively associated with hypertension (unpublished). However, due to cross-sectional study design, we cannot draw conclusions on nutritional longitudinal associations between dietary patterns and obesity/hypertension. Thus the aims of the present study were 1) to assess whether any changes exist in dietary patterns over seven years; 2) to elucidate the longitudinal associations in body mass index (BMI), weight (Wt), waist circumference (WC) and hypertension (Yes/No) with dietary patterns during seven years follow-up.

## Methods

### China Health and Nutrition Survey (CHNS)

CHNS is an ongoing open cohort longitudinal survey of nine waves (1989–2011). The survey uses a multistage random-cluster sampling process to select samples from nine provinces across China, which vary substantially in geography, economic development and health indicators. Details of CHNS sampling are described elsewhere [6, 10]. In 2004, 2 197 adults aged 60 years or older provided dietary information and physical measurements of weight, height, WC, and systolic and diastolic blood pressure. We followed up the participants in 2004, the number of participants were 1 763 in 2006, 1 303 in 2009 and 1085 in 2011, respectively. Total number of observations used in the present study was 6348.

### Dietary assessment and food grouping

Dietary assessment is based on each participant’s 24 hour-recall, with information being collected over three consecutive days. The three consecutive days during which detailed food consumption data have been collected were randomly allocated from Monday to Sunday. Over 99 % of the participants were available for all the 3 days dietary data. Details of the dietary data collection are described elsewhere [6, 10, 11].

We used a food grouping method in our previous report [3]. Initially, 33 food groups were included. As some food items were consumed by less than 5 % of participants, food intakes were further collapsed into 27 food groups based on similarity of nutritional profiles. The 27 food groups used are: rice; wheat flour and wheat noodles; wheat buns and bread; corn and coarse grains; deep-fried wheat; starchy roots and tubers; pork; red meat; organ meat; processed meats; poultry and game; fish and seafood; milk; eggs and egg products; fresh legumes; legume products; dried legumes; fresh vegetables, non-leafy; fresh vegetables, leafy; pickled, salted or canned vegetables; dried vegetables; cakes; fruits; nuts and seeds; beer; liquor; and fast food.

The average consumption per day from each food group was calculated from the dietary recall data. Intakes of food were converted onto Chinese ounces (liang; 1 liang = 50 g). For the alcoholic beverages, we calculated intake from the response of the questions on drinking frequency, types and quantity consumed in a week. The details are described in our previous report [3].

### Outcome variables

Height, body weight and WC were measured based on a standard protocol recommended by the World Health Organization (WHO). Each participant was weighed in lightweight clothing, with the measurement taken on a calibrated beam scale, and the weight recorded to the nearest 0.01 kg. Height was measured without shoes using a portable stadiometer, and recorded to the nearest 0.1 cm [10]. We calculated the BMI as weight in kilograms divided by the square of the height in meters [12]. Hypertension was defined by combining systolic blood pressure(SBP) > 140 mmHg and/or diastolic blood pressure(DBP) > 90 mmHg, a self-reported diagnosis of hypertension, or by taking anti-hypertensive medication.

### Covariates

Socio-demographic factors included in the study are age, gender, marital status (married and others), work status (Yes/No), education (illiteracy; low: primary school; medium: junior middle school; and high: high middle school or higher) and urbanization levels (low, medium and high) [11, 13]. Health behaviour factors included smoking, drinking and physical activity levels. Smokers were identified as people who smoke at least one cigarette per day, based on the question ‘how many cigarettes do you smoke per day?’ Alcohol consumption was allocated to two categories (Yes/No), with the question ‘last year, did you drink beer or any other alcoholic beverage?’ We calculated Metabolic Equivalent of Task (MET) to identify physical activity level based on the Compendium of Physical Activities [14, 15].

### Statistical analysis

Dietary patterns derived by the intake(liang or cups) of 27 food groups were analysed using principal component analysis to identify explanatory factors [3]. The number of dietary patterns was identified based on the eigenvalue (>1), scree plot, factor interpretability and the variance explained (>5 %). Factors were rotated with varimax rotation to improve the interpretability of the factors and minimize the correlation between them. Factor loadings are equivalent to correlation between food items and factors. Higher loadings indicate a higher shared variance with the factor. Factor loadings of > |0.20| represent the foods that most strongly related to the identified factor [3]. We recognised two dietary patterns and assigned participants based on their pattern-specific factor score. We further predicted the scores for other survey years based on the factor solution in 2009.

Factor scores were divided into quartiles based on their distribution in each stratum, implying increased intake from quartile 1 (Q1) to quartile 4 (Q4). Mean and standard deviation across four quartiles were used to present the average BMI, Wt, WC, SBP and DBP in each quartile of each dietary pattern. Linear Mixed Models (LMM) were used to investigate associations of dietary patterns with BMI, WC, Wt, SBP and DBP (continuous variables). Marginal plots were used to present the interaction terms from the LMM. Generalized Estimating Equation models were used to assess the relationships between dietary pattern and hypertension (binary variable). Sensitivity analysis was conducted to investigate potential errors and their impacts on conclusions to be drawn from the models. All analyses were conducted in STATA/SE 13.1 (STATA, StataCorp, USA).

## Results

Table 1 shows the characteristics of study participants in 2004, 2006, 2009 and 2011. Significant differences were found between participants for different survey years in their physical activity, work status, marital status, education level and urbanization levels (*p* < 0.05).

**Table 1:** Characteristics of study participants in 2004, followed by 2006, 2009 and 2011

Factors	2004	2006	2009	2011	*P* value*
N	2197	1765	1304	1086	
Physical activity (MET)					
Median (IQR)	79.9 (63.9; 109.0)	78.9 (63.5; 102.1)	89.9 (68.4; 119.1)	91.1 (68.5; 119.9)	<0.001
Gender					
Men	1030 (46.9 %)	837 (47.7 %)	620 (47.5 %)	518 (47.7 %)	0.97
Women	1167 (53.1 %)	928 (52.6 %)	684 (52.5 %)	568 (52.3 %)	
Work Status					
Yes	521 (23.8 %)	396 (22.4 %)	267 (20.5 %)	218 (20.1 %)	0.04
No	1668 (76.2 %)	1369 (77.6 %)	1037 (79.5 %)	868 (79.9 %)	
Marital status					
Married	1553 (71.2 %)	1272 (72.3 %)	896 (68.8 %)	708 (65.3 %)	<0.001
Others marital status^a^	627 (28.8 %)	487 (27.7 %)	406 (31.2 %)	376 (34.7 %)	
Education levels					
Illiteracy	759 (34.7 %)	699 (39.9 %)	499 (38.4 %)	407 (37.7 %)	0.006
Low	859 (39.3 %)	602 (34.3 %)	502 (38.7 %)	411 (38.0 %)	
Medium	285 (13.0 %)	215 (12.3 %)	153 (11.8 %)	146 (13.5 %)	
High	285 (13.0 %)	238 (13.6 %)	144 (11.1 %)	117 (10.8 %)	
Smoking status					
Yes	538 (24.5 %)	392 (22.2 %)	320 (24.5 %)	266 (24.5 %)	0.30
No	1659 (75.5 %)	1373 (77.8 %)	984 (75.5 %)	820 (75.5 %)	
Urbanization levels					
Low	793 (36.1 %)	624 (35.4 %)	406 (31.1 %)	297 (27.4 %)	<0.001
Medium	701 (31.9 %)	516 (29.2 %)	466 (35.7 %)	431 (39.8 %)	
High	703 (32.0 %)	625 (35.4 %)	432 (33.1 %)	354 (32.7 %)	

*ANOVA tests were used to examine the association between survey years and gender, work status, marital status, education levels, smoking status, and urbanization levels. Linear regression was used to access the association between physical activity levels and survey years

^a^Other marital status includes divorced; widowed; separated and never married

Two dietary patterns were obtained from the factor analysis performed in our previous study [3]. Factor 1 (‘Traditional’) was loaded heavily on rice, pork and vegetables, and inversely on wheat flour and wheat buns. Factor 2 (‘Modern’) was characterised by high intake of dairy, fruit, cakes and fast food, and inversely on rice and wheat flour. The two factors explained 14.5 % of the variance in intake. We used the data on food intake from 2009 to derive the factors that identified the different dietary patterns [3, 16], and applied the factor loadings to each of the individuals' food intakes to generate factor scores for other survey years.

**Fig. 1:**
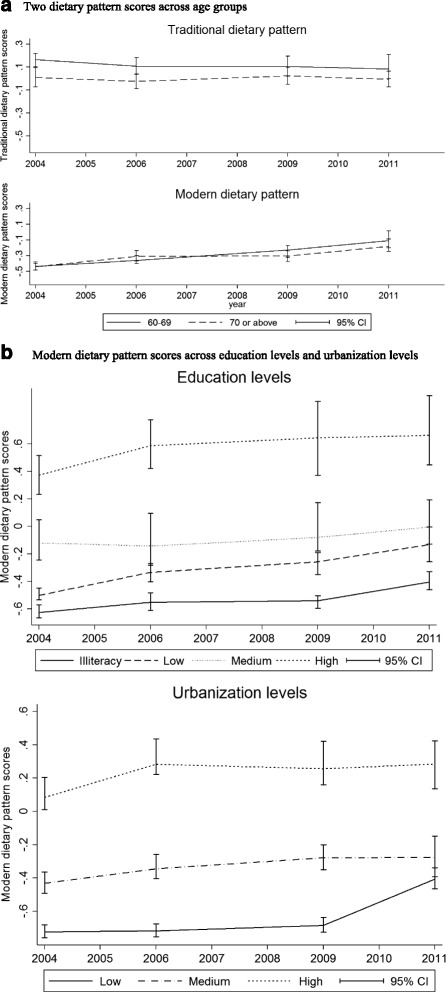
Dietary pattern scores transition across years. **a** Two dietary pattern scores across age groups. **b** Modern dietary pattern scores across education levels and urbanization levels

Figure 1 presents the dietary pattern scores transitions from 2004 to 2011, according to age groups, education levels and urbanization levels. Figure 1a shows that traditional dietary pattern scores decreased slightly or were stable, while modern dietary pattern scores increased over the years across age groups (*p* < 0.001). Figure 1b shows that compared with those with lower education level, participants with higher education level have higher modern dietary pattern scores; compared with those live in the low urbanization level, participants who live in the high urbanization level have higher modern dietary pattern scores.

Table 2 shows the BMI, Wt, WC, SBP and DBP changes by quartiles of dietary patterns in four survey years. A significant decrease in BMI was found for traditional dietary pattern in Q2 and modern dietary pattern in Q4 (p for trend = 0.004). A significant decrease in Wt was found for both dietary patterns, while a significant increase in WC was found for both dietary patterns. Significant increases in SBP were found, while DBP remained stable for both dietary patterns.

**Table 2:** BMI, WC and WHtR changes by quartiles of dietary patterns across four survey years

	Survey year				
	2004	2006	2009	2011	P for trend*
	BMI				
Traditional	Mean (s.d)	Mean (s.d)	Mean (s.d)	Mean (s.d)	
Q1	23.6 (3.8)	23.6 (3.9)	23.6 (3.8)	23.6 (4.0)	0.90
Q2	23.6 (4.0)	23.3 (4.0)	22.9 (4.0)	23.3 (4.0)	0.004
Q3	23.0 (3.6)	22.4 (3.5)	22.7 (3.7)	22.5 (3.6)	0.52
Q4	22.2 (3.4)	22.5 (3.5)	22.5 (3.7)	22.4 (3.5)	0.50
Modern					
Q1	22.0 (3.5)	22.1 (3.6)	22.1 (3.8)	21.9 (3.3)	0.15
Q2	22.7 (3.8)	22.3 (3.7)	22.3 (3.6)	22.6 (3.8)	0.33
Q3	23.7 (3.7)	23.4 (3.8)	23.3 (3.9)	23.2 (3.8)	0.06
Q4	24.2 (3.7)	24.1 (3.7)	24.0 (3.6)	23.6 (3.9)	0.004
	Weight				
Traditional					
Q1	60.0 (11.7)	59.4 (11.7)	59.5 (11.7)	59.3 (12.6)	0.01
Q2	58.5 (11.8)	57.4 (11.8)	56.1 (12.3)	56.6 (11.4)	<0.001
Q3	55.9 (11.0)	54.5 (10.5)	55.8 (11.2)	54.8 (11.1)	0.34
Q4	55.3 (10.9)	56.4 (11.0)	56.5 (11.1)	55.3 (10.9)	0.02
Modern					
Q1	53.5 (10.6)	53.1 (10.3)	53.1 (10.6)	51.8 (10.0)	0.04
Q2	55.4 (11.0)	53.6 (10.5)	54.2 (10.9)	54.0 (10.4)	0.37
Q3	59.1 (11.5)	58.7 (10.7)	58.3 (11.9)	57.5 (11.9)	0.001
Q4	62.8 (10.9)	62.1 (11.4)	61.5 (11.2)	60.1 (12.0)	<0.001
	WC				
Traditional					
Q1	84.4 (11.5)	84.7 (11.1)	86.1 (10.5)	86.2 (10.8)	<0.001
Q2	84.3 (11.0)	84.4 (10.5)	83.4 (10.9)	85.2 (10.5)	0.29
Q3	82.6 (10.3)	81.7 (10.0)	83.6 (10.9)	82.9 (10.7)	0.007
Q4	80.6 (10.3)	81.8 (10.6)	82.4 (10.5)	82.6 (10.7)	0.001
Modern					
Q1	80.8 (10.2)	80.6 (10.0)	81.4 (10.3)	81.3 (9.9)	0.004
Q2	81.2 (10.9)	80.9 (10.1)	82.4 (10.6)	83.2 (11.0)	<0.001
Q3	83.9 (11.5)	84.6 (10.9)	84.5 (11.1)	85.4 (10.6)	0.04
Q4	86.5 (10.0)	86.4 (10.6)	86.8 (10.3)	85.7 (10.7)	0.57
	Hypertension				
	SBP				
Traditional					
Q1	133.0 (21.7)	132.0 (20.0)	135.3 (19.0)	136.1 (18.7)	<0.001
Q2	136.0 (22.4)	132.7 (22.1)	137.0 (21.0)	136.1 (21.3)	0.05
Q3	133.7 (22.5)	130.9 (21.0)	136.8 (20.0)	135.8 (21.4)	0.002
Q4	133.4 (21.6)	132.2 (20.5)	135.8 (21.9)	135.3 (22.6)	0.008
Modern					
Q1	132.2 (23.9)	130.3 (21.4)	133.4 (19.8)	132.0 (24.1)	0.14
Q2	135.0 (22.1)	129.7 (20.7)	136.9 (22.2)	137.2 (20.4)	0.002
Q3	135.2 (21.7)	134.2 (20.8)	136.4 (19.3)	137.0 (19.3)	0.06
Q4	133.9 (19.6)	133.5 (20.5)	138.1 (20.3)	135.8 (20.9)	0.001
	DBP				
Traditional					
Q1	81.8 (12.4)	81.7 (11.4)	83.9 (11.8)	82.0 (10.1)	0.03
Q2	82.2 (13.3)	81.6 (13.0)	82.5 (12.2)	79.9 (11.8)	0.33
Q3	80.3 (12.0)	80.4 (12.2)	81.3 (11.3)	78.6 (12.4)	0.62
Q4	80.5 (11.5)	80.7 (11.3)	81.3 (11.5)	78.1 (11.5)	0.03
Modern					
Q1	81.4 (13.7)	80.2 (12.2)	82.3 (12.3)	77.0 (12.4)	0.20
Q2	80.8 (12.5)	79.5 (11.7)	81.6 (12.1)	80.7 (11.8)	0.19
Q3	81.8 (11.5)	82.6 (12.7)	82.6 (12.1)	79.7 (10.8)	0.16
Q4	80.7 (11.0)	82.0 (11.2)	82.4 (10.6)	80.4 (11.4)	0.92

* Linear regressions were used to examine the associations between both dietary patterns and BMI, weight, WC, SBP and DBP

Table 3 shows the associations between dietary patterns and BMI, Wt and WC. In the fully adjusted model (Adjusted^c^), the traditional dietary pattern was significantly inversely associated with BMI, Wt and WC. Using the first quartile as the reference, participants in the highest quartile of traditional dietary pattern had a β (difference in mean) of −0.23 (95 % CI: −0.44; −0.02) for BMI decrease, β of −0.90 (95 % CI: −1.42; −0.37) for Wt decrease, and β of −1.57 (95 % CI: −2.32; −0.83) for WC decrease.

**Table 3:** The association between dietary pattern and BMI, weight and waist circumference

	Quartiles of dietary pattern							
	Q1	Q2		Q3		Q4		P for trend
	Ref	β	95 % CI	β	95 % CI	β	95 % CI	
Traditional	BMI							
Unadjusted	0	0.04	−0.14; 0.21	−0.08	−0.28; 0.12	−0.05	−0.26; 0.15	0.43
Adjusted^a^	0	−0.02	−0.20; 0.15	−0.17	−0.37; 0.03	−0.23	−0.44; −0.02	0.02
Adjusted^c^	0	−0.03	−0.20; 0.15	−0.17	−0.37; 0.02	−0.23	−0.44; −0.02	0.02
	Weight (kg)							
Unadjusted	0	−0.01	−0.44; 0.43	−0.22	−0.71; 0.28	0.03	−0.49; 0.54	0.89
Adjusted^a^	0	−0.31	−0.75; 0.12	−0.76	−1.26; −0.27	−0.88	−1.41; −0.36	0.001
Adjusted^c^	0	−0.33	−0.76; 0.11	−0.78	−1.27; −0.28	−0.90	−1.42; −0.37	0.001
	WC (cm)							
Unadjusted	0	−0.58	−1.21; 0.05	−0.87	−1.57; −0.17	−1.35	−2.08; −0.62	<0.001
Adjusted^a^	0	−0.67	−1.30; −0.03	−1.02	−1.72; −0.32	−1.57	−2.32; −0.83	<0.001
Adjusted^c^	0	−0.67	−1.30; −0.03	−1.01	−1.71; −0.31	−1.57	−2.32; −0.83	<0.001
Modern	BMI							
Unadjusted	0	0.15	0.02; 0.29	0.25	0.10; 0.40	0.33	0.16; 0.50	<0.001
Adjusted^b^	0	0.18	0.04; 0.32	0.27	0.11; 0.42	0.29	0.12; 0.47	0.001
Adjusted^c^	0	0.18	0.04; 0.32	0.27	0.11; 0.42	0.29	0.12; 0.47	0.001
	Weight (kg)							
Unadjusted	0	0.31	−0.03; 0.65	0.49	0.11; 0.86	0.87	0.45; 1.28	<0.001
Adjusted^b^	0	0.50	0.15; 0.85	0.69	0.31; 1.07	1.01	0.57; 1.45	<0.001
Adjusted^c^	0	0.51	0.16; 0.86	0.70	0.32; 1.09	1.02	0.58; 1.46	<0.001
	WC (cm)							
Unadjusted	0	0.54	0.01; 1.07	1.58	1.01; 2.15	2.29	1.67; 2.91	<0.001
Adjusted^b^	0	0.35	−0.19; 0.88	1.10	0.51; 1.68	1.44	0.78; 2.10	<0.001
Adjusted^c^	0	0.35	−0.19; 0.89	1.10	0.51; 1.68	1.44	0.78; 2.10	<0.001

Adjusted^a^ model was adjusted for age, urbanization, gender, marital status, work status, education level, smoking, physical activity, modern dietary pattern and energy; Adjusted^b^ model was adjusted for age, urbanization, gender, marital status, work status, education level, smoking, physical activity, traditional dietary pattern and energy intake. Adjusted^c^ was adjusted for other NCDs (known diabetes, myocardial infarction and stoke) and Adjusted^a^ (or adjusted^b^) model

By contrast, modern dietary pattern showed significant positive associations with BMI, Wt and WC. Participants in the highest quartile of the modern dietary pattern had a β of 0.29 (95 % CI: 0.12; 0.47) for BMI increase; β of 1.02 (95 % CI: 0.58; 1.46) increase, and β of 1.44 (95 % CI: 0.78; 2.10) for WC increase.

**Fig. 2:**
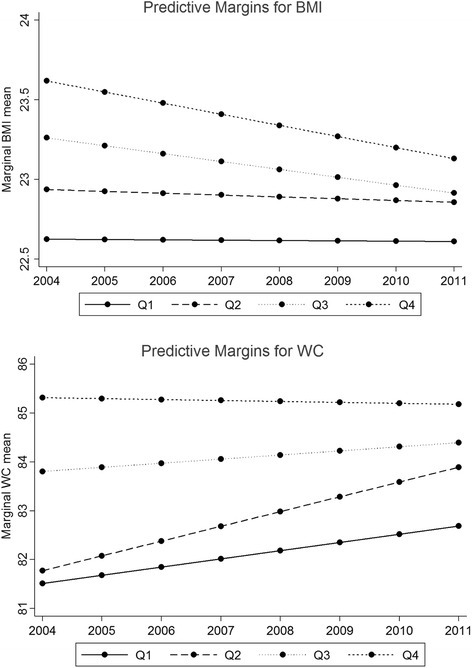
Predictive margins of quartiles of modern dietary pattern across years. *Marginal plot after adjustment for baseline age, urbanization, gender, marital status, work status, education level, smoking, physical activity, traditional dietary pattern, energy, other NCDs, and interaction between survey year and modern dietary pattern

The interactions were found for BMI/WC according to modern dietary pattern and survey years. Figure 2 shows the predictive margins of quartiles of modern dietary pattern across years. The BMI mean was decreasing with time for Q3 and Q4 of modern dietary pattern, while it stayed stable during this 7-year period for Q1 and Q2. By contrast, we observed a large increase in WC during this 7-year period in Q1 and Q2, while WC remained stable in Q3 and Q4.

Table 4 shows the association between dietary patterns and hypertension. In the adjusted model, no significant differences were found for both dietary patterns (Adjusted^a^ and Adjusted^b^). The association between traditional dietary pattern and hypertension reversed and became significant by adjusting for WC and BMI (p for trend < 0.05).

**Table 4:** The association between dietary pattern and hypertension

	Quartiles of dietary pattern							
	Q1	Q2		Q3		Q4		P for trend
	Ref	OR	95 % CI	OR	95 % CI	OR	95 % CI	
Traditional								
Unadjusted	1	1.08	0.93; 1.24	0.94	0.81; 1.09	0.92	0.78; 1.07	0.93
Adjusted^a^	1	1.06	0.91; 1.23	0.94	0.80; 1.10	0.94	0.80; 1.12	0.66
Adjusted^a^ + BMI	1	1.12	0.96; 1.32	1.04	0.88; 1.23	1.09	0.92; 1.30	0.04
Adjusted^a^ + WC	1	1.12	0.95; 1.31	1.02	0.86; 1.20	1.07	0.90; 1.28	0.04
Modern								
Unadjusted	1	1.14	1.00; 1.30	1.31	1.14; 1.50	1.32	1.14; 1.52	<0.001
Adjusted^b^	1	1.05	0.91; 1.20	1.20	1.03; 1.40	1.08	0.91; 1.27	0.29
Adjusted^b^ + BMI	1	1.01	0.88; 1.17	1.13	0.97; 1.33	1.00	0.84; 1.19	0.79
Adjusted^b^ + WC	1	1.08	0.93; 1.24	1.18	1.01; 1.38	1.03	0.87; 1.23	0.78

Adjusted^a^ model was adjusted for age, urbanization, gender, marital status, work status, education level, smoking, physical activity, modern dietary pattern, energy, salt, and other NCDs; Adjusted^b^ model was adjusted for age, urbanization, gender, marital status, work status, education level, smoking, physical activity, traditional dietary pattern, energy intake, salt and other NCDs

### Sensitivity analysis

Based on participants in 2011 (*N* = 1085), we followed back the same participants in 2004 to examine the dietary patterns scores transitions, and the associations between dietary patterns and BMI, Wt, WC and hypertension among the same population. Additional file 1 shows that the mean of the traditional dietary pattern scores dramatically decreased from 0.09 to −0.07, while the mean of the modern dietary pattern scores dramatically increased from −0.24 to 0.13. Compared with results we presented above, the direction of dietary pattern scores transition, also the association remained the same (Additional file 2).

In order to assess bias, we compared the baseline participants (*N* = 2197 in 2004) and participants in the final wave (*N* = 1085 in 2011). During the survey period, 289 participants died, and 823 participants were lost to follow up. We compared the baseline factor scores according to three categorical groups (death; lost to follow-up and follow-up participants). The marginal mean of dietary patterns factor scores at baseline are shown in the Additional file 3. Participants who were lost to follow-up have higher modern dietary pattern scores.

## Discussion

The present 7-year longitudinal study shows that over time, older people’s diet has shifted towards the modern dietary pattern, and people with higher education level, and individuals living in the high urbanization level were more likely to have more modern diet. We found this change over time is consistent with secular trend, regardless of age, and contrary to the exception that people’s diets became more traditional as their age. In addition, the modern dietary pattern was associated with an increase in BMI, weight and WC, whereas the traditional dietary pattern led to a decrease in BMI, weight and WC. In this analysis we used the data from one survey to determine the dietary patterns as it was our intention to hold the definition of a traditional diet constant over time.

From 2004 to 2011, BMI and Wt were slightly decreasing over the years. This is mainly due to ageing being associated with a change in body composition, such as reduced amount of lean body mass [5]. Loss of muscle and thus strength contributes to functional impairment that can further developing in sarcopenia among older population [17]. Additionally, BMI can be affected in the older population as they tend to shrink with age, with loss of bone mass or density being the main reason for weight loss [18]. WC increased with age, from 82.9 cm in 2004 and 84.3 cm in 2011 (p for trend <0.001). As the BMI did not change much for older population, while WC largely increased over the years, this may suggest that BMI is an inferior predictor for NCDs. There is strong emerging evidence that WHO cut-offs for BMI may not be appropriate in increasing age [19, 20]. By meta-analysis of 32 longitudinal studies, Winter et al. shows that older people (≥65 years) who stand at the lower end of the recommended BMI range, have an increased the risk of mortality, while for those being overweight there was no increased risk of mortality [20]. Another longitudinal study shows that BMI was not associated with NCDs, while WC was strong associated with conditions, such as chronic heart failure [21] in the older population. The increased WC observed in the present study, needs to be addressed, as obesity in the abdominal area is associated with risk of metabolic syndrome [22] and higher mortality [20].

Our study found similar results to Batis et al. studies [23, 24] which also undertook longitudinal analysis of CHNS data, and found the increasing popularity of the modern dietary pattern. Our study adds to these by focussing on the older Chinese population, with our results showing that modern dietary pattern scores have dramatically increased during survey years among people aged 60 or above Reasons for this may lie in the dietary transitions due to shifts in the agricultural system and subsequent growth of modern retail and food service sectors in China in recent decades. These shifts in diet are towards increased refined carbohydrates, added sweeteners, edible oils, food from animal-sources, and decreased intake in legumes, fruit and vegetables [25]. Additionally, we found that this modern dietary pattern was preferred by people with higher education, and individuals living in the high urbanization level. Our previous study shows that older people with high education level have higher relative fat intakes (energy from fat) than those with illiterate, low or medium education levels; and people living in areas of high urbanization have higher relative fat intakes than those living in low- and medium urbanization levels [6]. Higher relative fat intake can partly explain the higher modern dietary pattern scores within these groups.

Some other studies have been conducted to assess changes in dietary pattern over time. Analysis of data from 33,840 women participating in the Swedish Mammography Cohort in 1987 and 1997, shows that changes in dietary patterns were significantly related to changes in BMI over nine years of follow up [26]. By using sequence analysis of 3418 participants at baseline in Framingham Heart Study, Pachucki [27] shows that adults with unhealthful trajectory are 1.79 times more likely to be overweight, and 2.4 times more likely to be obese. These results are consistent with our study, finding the strong associations between favourable body composition and traditional diet (health diet), compared with an increase in BMI, WC and Wt with modern diet (unhealthy diet).

The present study confirms our previous cross-sectional findings of a relationships between dietary patterns and obesity [3]. The traditional diet with its main components of rice, pork, fish and vegetables contributes to the inverse association with BMI, WC and weight. This is opposed by the modern diet with main components being processed and fast foods and a positive association with BMI, WC and weight. Although there is still dispute about the role of a rice-based dietary pattern in preventing obesity in Asian countries [28–30], we found a diet with a high proportion of rice and vegetables helps to prevent weight gain, large WC and obesity in China. Rice is a low-energy food [29] and the predominant component of the traditional dietary pattern, but contributes little to modern dietary pattern.

Interestingly, although the modern dietary pattern contains too much fat, which contributes to the positive association with BMI/WC, BMI of older Chinese decreasing with the length of time were followed. This suggests the modern dietary pattern is a key player in age-related loss of muscle and bone mass. The increase in WC for a modern dietary pattern is consistent with current knowledge. Fat is redistributed from subcutaneous to intra-abdominal visceral depots during and after middle age. In old age, fat is redistributed to bone marrow, muscle, liver, and other ectopic sites. Also, the percent of ingested fat that is stored in subcutaneous depots is lower in older than young people, and the abdominal circumference increases in the old age [31]. Further research is require to identify the specific components of the modern dietary pattern, which can lead to loss of muscle and bone mass. Although we did not find significant associations between dietary patterns and hypertension, we found BMI and WC are potential confounding or matching variables for hypertension.

The shift in dietary pattern over the years towards a modern diet is associated with rapid economic and social development in China. Older people have specific dietary needs and are at high risk of an unbalanced diet, which suggests that dietary guidelines should be developed for the older population. Although there is general advice for people aged 60 years or over [11, 32], age-specific guidelines for the older population is extremely important to encourage healthy eating in promoting healthy ageing, especially within the context of the aging Chinese population.

The strengths of the present study include the use of individual 24 h recall over consecutive 3 days. This method improves the accuracy of recall and hence analysis and results, and four time points allowing longitudinal analysis of associations. A weakness of this study is the large amount of missing data due to attrition. For continuous outcomes we analysed the data using LMMs, which are valid under the assumption that, conditional on the covariates included in the model, the data are missing at random. For the dichotomous outcome of hypertension we used the generalised estimating equation framework, which are valid under the assumption that the data are missing completely at random, conditional on the covariates included in the models. In our most comprehensive models we included the covariates of age, urbanization, gender, marital status, work status, education level, smoking, physical activity, traditional dietary pattern and energy intake, known diabetes, myocardial infarction and stoke. However, it’s possible and even probable that after taking these variables into account there are unmeasured characteristics which predict missingness and hence the data would be considered missing not at random. We could have used multiple imputations to impute the missing data under some assumptions about the missing data but the likelihood of getting the appropriate mechanism correct is low, and therefore we do not believe that it would have added anything to the analysis. With participants lost to follow-up more likely to be from the high risk group (Additional files 1, 2 and 3, Fig. 2), the GEE may underestimate the associations between dietary patterns and BMI, Wt, WC and hypertension. However, when we fit a random effects logistic regression model to the hypertension outcome we get very similar *p*-values to those from the GEE, but we have chosen to report the results from the GEE in this paper because we believe the population average interpretation is more appropriate in this circumstance.

The potential limitation is due to measurement error of food intake levels, residual confounding and the relatively short follow-up time. Some studies show that different types of rice result in different glycaemic responses, and their consumption may affect dietary management of obesity [33] (such as brown rice have beneficial role than white rice), we are not able to distinguish the effects of each type of rice as the consumption of brown rice by the study participants is low.

## Conclusions

The present 7-year longitudinal study leads to the conclusion that a rice-based traditional dietary pattern can lead to lower weight, BMI and WC in old age; while the modern dietary pattern can lead to increase in weight, BMI and WC. This study is particularly important in the context of China’s ageing population and has implications for nutritional interventions, planning and policies in prevention obesity and NCDs for older people in China.

## Abbreviations

BMI, body mass index; CHNS, China Health and Nutrition Survey; DBP, diastolic blood pressure; LMM, linear mixed models; NCDs, non-communicable diseases; SBP, systolic blood pressure; WC, waist circumference; WHO, World Health Organization; Wt, weight

## Additional files

Additional file 1: Factor scores transition in 2004 and 2011 (*N*=1085). (PDF 86 kb) Additional file 2: The association between dietary pattern and BMI, Wt, WC and hypertension for participants in 2004 and 2011. (PDF 179 kb) Additional file 3: Marginal mean of dietary patterns at baseline by three groups (*N*=2197). (PDF 125 kb)
