# Supplementary figures and images for: Dietary pattern transitions, and the associations with BMI, waist circumference, weight and hypertension in a 7-year follow-up among the older Chinese population: a longitudinal study
Source: BMC Public Health. 2016 Aug 8;16:743. doi: 10.1186/s12889-016-3425-y (PMC4977626; doi:10.1186/s12889-016-3425-y)

**Additional file 1.**

**Factor scores transition in 2004 and 2011 (N=1085)**

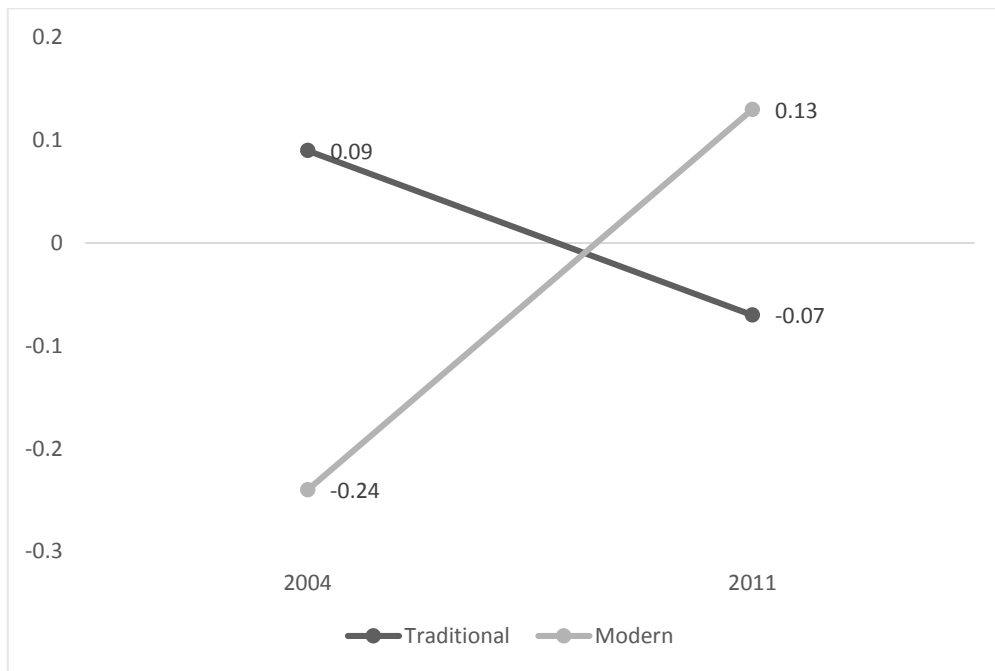

Supplement: Additional file 1: — Factor scores transition in 2004 and 2011 (N=1085). (PDF 86 kb) [file 12889_2016_3425_MOESM1_ESM.pdf]
