# Supplementary material for: Dietary pattern transitions, and the associations with BMI, waist circumference, weight and hypertension in a 7-year follow-up among the older Chinese population: a longitudinal study
Source: BMC Public Health. 2016 Aug 8;16:743. doi: 10.1186/s12889-016-3425-y (PMC4977626; doi:10.1186/s12889-016-3425-y)
Supplement: Additional file 2: — The association between dietary pattern and BMI, Wt, WC and hypertension for participants in 2004 and 2011. (PDF 179 kb) [file 12889_2016_3425_MOESM2_ESM.pdf]

Additional file 2.

The association between dietary pattern and BMI, Wt, WC and hypertension for participants in 2004 and 2011

	Quartiles of dietary pattern							
	Q1		Q2		Q3		Q4	P for trend
	Ref	OR	95% CI	OR	95% CI	OR	95% CI	
				BMI				
Traditional								
Adjusted[a]	1	0.04	-0.31; 0.40	-0.32	-0.70; 0.07	-0.65	-1.06; 0.25	<0.001
Modern								
Adjusted[b]	1	0.30	-0.01; 0.61	0.65	0.33; 0.98	0.44	0.07; 0.81	0.005
				Wt				
Traditional								
Adjusted[a]	1	-0.82	-1.72; 0.09	-1.68	-2.67; -0.69	-2.55	-3.60; -1.49	<0.001
Modern								
Adjusted[b]	1	0.60	-0.17; 1.36	1.70	0.88; 2.52	1.71	0.77; 2.64	0.02
				WC				
Traditional								
Adjusted[a]	1	-0.50	-1.67; 0.66	-1.04	-2.26; 0.19	-2.35	-3.63; -1.07	<0.001
Modern								
Adjusted[b]	1	0.55	-0.50; 1.61	1.58	0.46; 2.71	1.79	0.54; 3.04	<0.001
				Hypertension				
Traditional								
Adjusted[c]	1	1.11	0.85; 1.46	1.05	0.79; 1.38	1.16	0.86; 1.58	0.09
Adjusted[c] + BMI	1	1.13	0.85; 1.49	1.15	0.86; 1.53	1.43	1.04; 1.96	0.002
Adjusted[c] + WC	1	1.14	0.86; 1.50	1.13	0.85; 1.51	1.41	1.03; 1.92	0.003
Modern								
Adjusted[d]	1	1.13	0.87; 1.46	1.46	1.11; 1.92	1.07	0.79; 1.45	0.29
Adjusted[d] + BMI	1	1.07	0.82; 1.39	1.31	0.99; 1.73	0.98	0.72; 1.33	0.67
Adjusted[d] + WC	1	1.11	0.85; 1.44	1.33	1.00; 1.75	0.98	0.72; 1.33	0.64

Adjusted[a] model was adjusted for age, urbanization, gender, marital status, work status, education level, smoking, physical activity, modern dietary pattern, energy, and other NCDs; Adjusted[b] model was adjusted for age, urbanization, gender, marital status, work status, education level, smoking, physical activity, traditional dietary pattern, energy, and other NCDs. Adjusted[c] model was adjusted for age, urbanization, gender, marital status, work status, education level, smoking, physical activity, modern dietary pattern, energy, salt, lead, and other NCDs; Adjusted[d] model was adjusted for age, urbanization, gender, marital status, work status, education level, smoking, physical activity, traditional dieary pattern, energy, salt and other NCDs.
