# Supplementary material for: Dietary pattern transitions, and the associations with BMI, waist circumference, weight and hypertension in a 7-year follow-up among the older Chinese population: a longitudinal study
Source: BMC Public Health. 2016 Aug 8;16:743. doi: 10.1186/s12889-016-3425-y (PMC4977626; doi:10.1186/s12889-016-3425-y)
Supplement: Additional file 3: — Marginal mean of dietary patterns at baseline by three groups (N=2197). (PDF 125 kb) [file 12889_2016_3425_MOESM3_ESM.pdf]

Additional file 3.

Marginal mean of dietary patterns at baseline by three groups (N=2197)

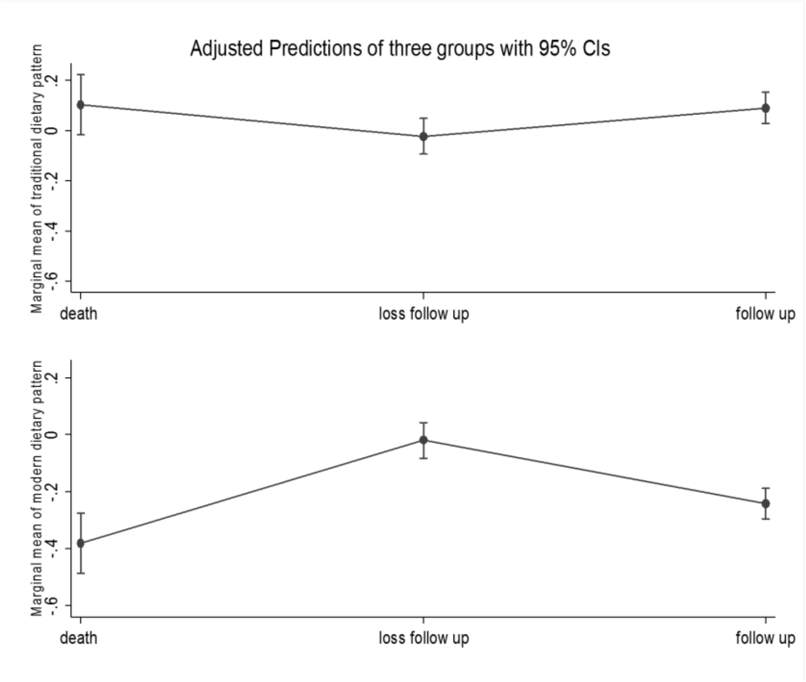

** Death: N=289; Lost to follow-up: N=823; and follow-up participants: N=1085.*
